# Subacute cutaneous lupus erythematosus as a rare complication of disease-modifying therapy administration in multiple sclerosis: case report

**DOI:** 10.1186/s12883-023-03146-1

**Published:** 2023-04-26

**Authors:** Ke Xu, Mengjie Zhang, Shilin Yang, Gang Yu, Peng Zheng, Xinyue Qin, Jinzhou Feng

**Affiliations:** 1 Department of Neurology, The First Affiliated Hospital of Chongqing Medical University, Chongqing, China; 2 National Health Commission Key Laboratory of Diagnosis and Treatment On Brain Functional Diseases, The First Affiliated Hospital of Chongqing Medical University, Chongqing, China; 3 Department of Neurology, Huashan Hospital, Fudan University, Shanghai, China

**Keywords:** Multiple sclerosis, Subacute cutaneous lupus erythematosus, Teriflunomide, Leflunomide, Autoimmune diathesis, Case report

## Abstract

**Background:**

Teriflunomide, the active metabolite of leflunomide, is a disease-modifying therapy drug used for the treatment of multiple sclerosis (MS), yet the complications associated with this drug remain not fully understood. Here we present the rare case of a 28-year-old female MS patient who developed subacute cutaneous lupus erythematosus (SCLE) following teriflunomide treatment. Though SCLE has been reported to be associated with leflunomide, the current report represents the first documented evidence demonstrating SCLE as a potential teriflunomide treatment-related complication. Additionally, a literature review on the leflunomide-induced SCLE was conducted to emphasize the association of SCLE with teriflunomide, specifically amongst the female demographic with a preexisting autoimmune diathesis.

**Case presentation:**

A 28-year-old female first presented with MS symptoms in the left upper limb along with blurred vision in the left eye. Medical and family histories were unremarkable. The patient exhibited positive serum biomarkers including ANA, Ro/SSA, La/SSB, and Ro-52 antibodies. Relapsing–remitting MS was diagnosed according to the 2017 McDonald’s diagnostic criteria, and remission was achieved upon intravenous administration of methylprednisolone followed by teriflunomide sequential therapy. Three months post-teriflunomide treatment, the patient developed multiple facial cutaneous lesions. SCLE was subsequently diagnosed and was attributed to treatment-related complication. Interventions include oral administration of hydroxychloroquine and tofacitinib citrate effectively resolved cutaneous lesions. Discontinuation of hydroxychloroquine and tofacitinib citrate treatment led to recurring SCLE symptoms under continuous teriflunomide treatment. Full remission of facial annular plaques was achieved after re-treatment with hydroxychloroquine and tofacitinib citrate. The patient’s clinical condition remained stable in long-term outpatient follow-ups.

**Conclusions:**

As teriflunomide has become a standard disease-modifying therapy for MS, the current case report highlights the importance of monitoring treatment-related complications, specifically in relation to SCLE symptoms.

## Background

Subacute cutaneous lupus erythematosus (SCLE) is a rare comorbidity related to several pharmacological and chemotherapy agents, such as hydrochlorothiazide [1], terbinafine [2], and calcium blockers [3]. SCLE presents clinically with erythematous annular or papulosquamous cutaneous eruptions in sun-exposed areas [4]. A total of 9 patients with autoimmune diseases who developed SCLE after receiving leflunomide have been reported thus far [5–12]. Teriflunomide, the active metabolite of leflunomide, has been approved as a disease-modifying therapy (DMT) drug for multiple sclerosis (MS) [13]. Drug-related complications include alopecia/hair thinning, liver enzyme elevation, diarrhea, and nausea [14]. However, no clinical studies thus far have reported SCLE as a potential drug-related adverse reaction of teriflunomide treatment. Here, we describe the first clinical case demonstrating a potential drug-related association between SCLE and teriflunomide treatment for MS.

## Case presentation

**Fig. 1:**
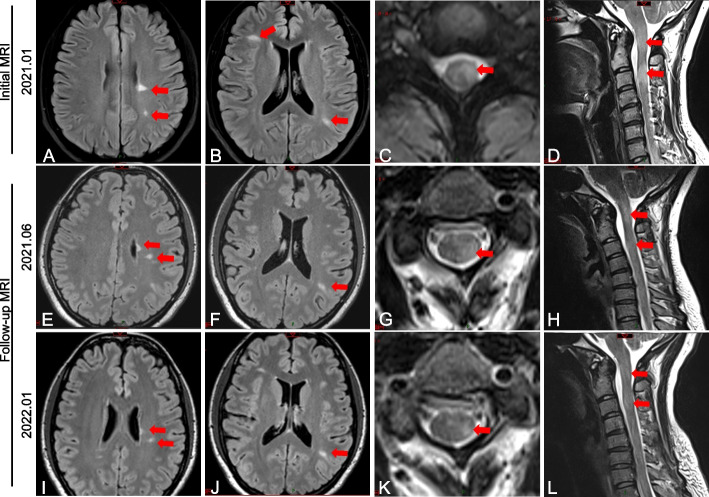
Initial brain and spine MRI of the axial sequence revealing multiple hyperintense lesions at the right anterior part of the upper pons and the left optic nerve (**A**) and the periventricular and subcortical matter (**B**) on FLAIR with hyperintense lesions in the left portion part of the cervical spinal cord (**C, D**). Repeated MRI showed a gradually decrease hyperintensity of periventricular, subcortical matter, and cervical spinal cord lesions after 5 months (**E–H**) and 1 year (**I-L**) after teriflunomide treatment

A 28-year-old female initially experienced right lower limb numbness that gradually spread to the left side of the body in April 2020. Three months later, limb numbness had completely recovered, except for in the left upper limb. In January 2021, the patient developed blurred vision in the left eye. No other vision-related symptoms including itching, discomfort, foreign-body sensation, burning/stinging, photophobia, tearing, and discharge were reported. Furthermore, arthralgias or myalgias were not noted. Her medical and family histories were unremarkable. Upon examination, the patient exhibited a nasal visual field defect of the left eye, left upper limb hypoalgesia, left knee hyperreflexia (+ + +), and a positive left Babinski sign. Left eye visual acuity was 20/200, and right eye visual acuity was 20/20. Lumbar puncture revealed positive cerebrospinal fluid-specific oligoclonal bands. No erythema, edema, or sclerotic skin plaque had been found on her limbs or trunk. Serum immunological tests showed that anti-nuclear antibody (ANA), and anti-extractable nuclear antigen (ENA), including anti-SSA (Ro), SSB (La), and Ro-52 antibodies were positive, while other laboratory tests (complete blood cell count, differential cell count, hepatic and thyroid function, urinalysis results and proteinuria, anti-mitochondrial antibody (AMA)/anti-liver-kidney microsomal antibody (LKM), anti-smooth muscle antibody (ASMA), antineutrophil cytoplasmic antibody (ANCA), erythrocyte sedimentation rate (ESR), C-reactive protein (CRP), anti-double stranded DNA antibody (dsDNA), complement C3, complement C4, serum immunoglobulin A (IgA), IgG, and IgM) were normal. The rheumatic factor was negative. Meanwhile, the patient received a consultation with rheumatologists and underwent a structured examination, including ultrasound examinations of the salivary, thyroid, and lacrimal glands. Considering that the patient presents no obvious clinical symptoms related to systemic autoimmune disease and abnormal glandular function, and only positive serum ANA, anti-Ro/SSA, La/SSB, and Ro-52 antibodies, rheumatologists suggest that the possibility of systemic autoimmune disease can be ruled out. Furthermore, brain magnetic resonance imaging (MRI) showed multiple hyperintense lesions in the left optic nerve, periventricular, and juxtacortical regions (Fig. 1A, B). An MRI of the cervical spinal cord showed gadolinium-enhanced lesions (Fig. 1C, D). The patient was subsequently diagnosed with relapse-remitting MS according to McDonald criteria 2017 [15] with an Expanded Disability Status Scale (EDSS) score of 3 points (vision, 4; sensory, 1; pyramidal functional system, 2).

**Fig. 2:**
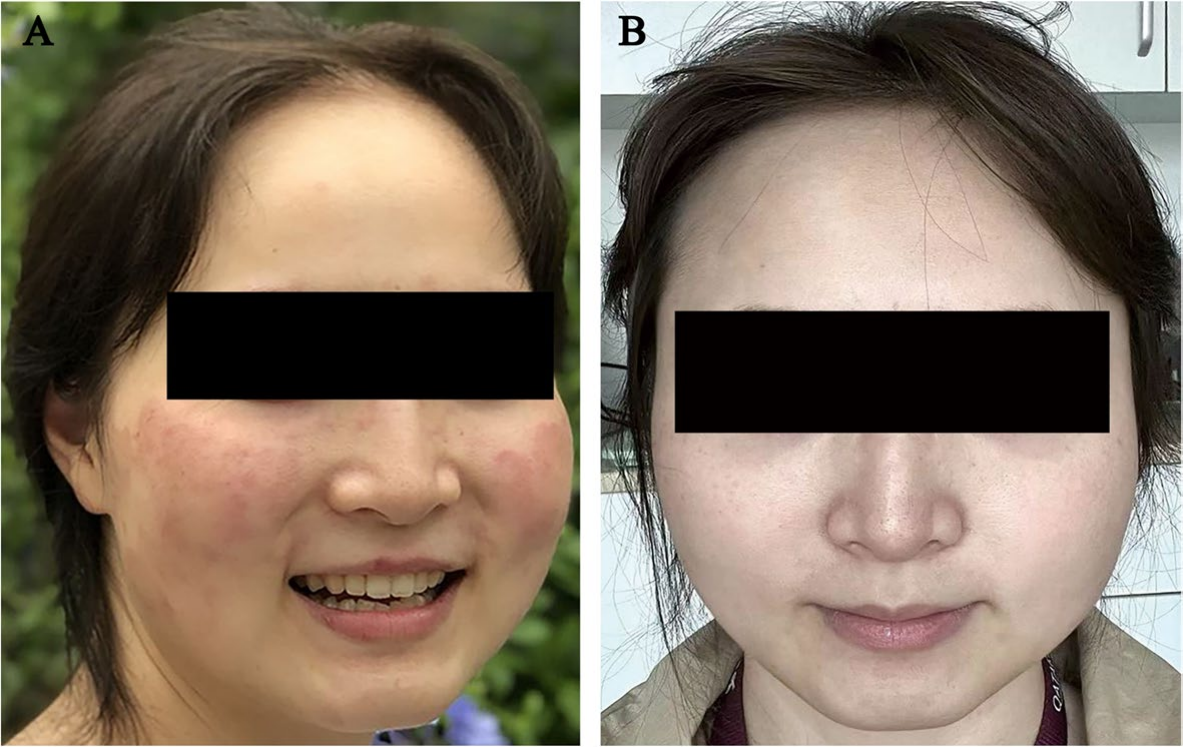
Multiple well-demarcated, painful, itchy erythematous annular lesions after using teriflunomide for three months (**A**), while significant alleviation was observed 6 weeks after treatment with hydroxychloroquine and tofacitinib citrate (**B**)

A significant recovery, indicated by a reduction of the EDSS score to 1 point (vision = 1), was observed after the intravenous administration of methylprednisolone at 1000 mg daily for 3d and 500 mg daily for 2d. In February 2021, the patient started daily oral administration of teriflunomide at 14 mg as a sequential DMT treatment. Three months post-teriflunomide treatment, multiple well-demarcated, painful, itchy erythematous annular lesions forming confluent patches begun to appear in the facial region. Symptoms exacerbated upon sun exposure (Fig. 2A). Laboratory test results indicated positive anti-Ro/SSA, anti-La/SSB, and anti-histidyl antibody (Jo-1). Additional laboratory tests include complete blood cell count, differential cell count, hepatic and thyroid function, urinalysis results and proteinuria, anti-AMA/anti-LKM, anti-ASMA, ANCA, ESR, CRP, anti-dsDNA, complement C3, complement C4, serum IgA, IgG, and IgM were normal. Facial skin fungal examination ruled out the possibility of fungal mycelia infection. Confocal laser scanning microscopy examination of the skin lesions showed that the basal layer was infiltrated with various amounts of dendritic cells, the dermal papilla and superficial capillaries were dilated and congested, and the perivascular infiltration of inflammatory cells was also observed. The clinical and serological features did not meet the diagnostic criteria of the American College of Rheumatology Classification Standard for SLE (2009) [16], but were consistent with the diagnosis of SCLE [17]. The dermatologist prescribed hydroxychloroquine 0.2 mg daily as an intervention strategy for SCLE. In addition, dermatologists consider that Janus kinase (JAK) is a key mediator involved in the pathogenesis of cutaneous lupus erythematosus, and its inhibitor tofacitinib has been used as a new strategy for treating cutaneous lupus erythematosus and related diseases [18]. Tofacitinib citrate tablets of 5 mg daily were also used. After 6 weeks of treatment, the patient’s symptoms and cutaneous lesions gradually resolved (Fig. 2B). The pathophysiology underlying of the SCLE development was attributed to the continued use of teriflunomide.

**Fig. 3:**
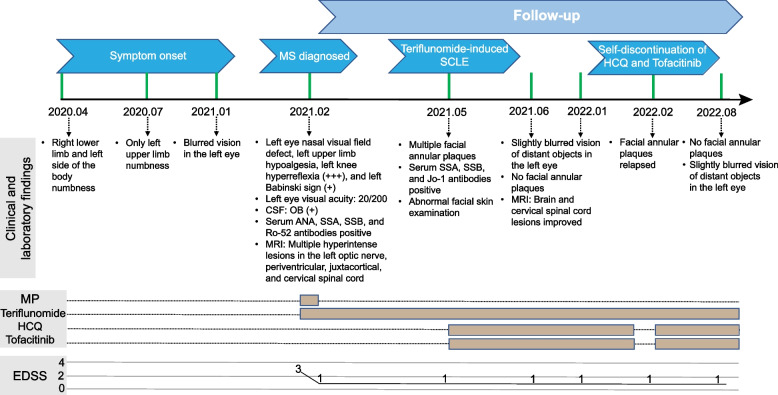
Case report timeline. *CSF*, cerebrospinal fluid; *EDSS*, expanded disability status scale; *HCQ*, hydroxychloroquine; *MRI*, magnetic resonance imaging; *MP*, methylprednisolone; *MS*, multiple sclerosis; *OB*, oligoclonal bands; *SCLE*, Subacute cutaneous lupus erythematosus

In February 2022, considering that the cutaneous lesions had subsided, the patient voluntarily stopped hydroxychloroquine and tofacitinib citrate. The facial annular plaques relapsed 2 weeks later. Laboratory test results also indicated positive anti-Ro/SSA, anti-SSB, and anti-Jo-1, while other tests (complete blood cell count, differential cell count, hepatic and thyroid function, urinalysis results and proteinuria, anti-AMA/anti-LKM, anti-ASMA, ANCA, ESR, CRP, anti-dsDNA, complement C3, complement C4, serum IgA, IgG, and IgM) were still normal. Reintroduction of hydroxychloroquine and tofacitinib citrate combo therapy as SCLE-related intervention completely resolves the plaque. In addition, clinical follow-up in June 2021 and January 2022, MRI of the brain and cervical spinal cord (Fig. 1E-L) showed no significant increase in lesion number and size longitudinally along with stable maintenance of EDSS score of 1. During the last follow-up in August 2022, the patient remained stable with a minimal blurred vision of distant objects in the left eye and an EDSS score of 1 with no clinical relapse during teriflunomide treatment. No facial annular plaques were observed. The case report timeline is shown in Fig. 3.

## Discussion and conclusions

This report presented a rare case of SCLE in a young female MS patient three-month post-treatment of teriflunomide at 14 mg dosage. To the best of our knowledge, this is the first reported case of SCLE associated with the use of teriflunomide. SCLE is recognized as a subtype of cutaneous lupus erythematosus that can be triggered by exogenous or endogenous factors, such as some drugs, tobacco, infections, and vaccines [19]. In particular, drug-induced SCLE exhibits clinical presentation of skin lesions mainly in sun-exposed areas. Although approximately 50% of patients with SCLE meet the criteria for systemic lupus erythematosus (SLE), patients with SLE were more likely than those with only SCLE to have positive anti-dsDNA antibody and ANA test findings. More recently, a study reported similar clinical symptoms where a subset of SCLE is characterized primarily by cutaneous disease along with the presence of anti-SSA antibodies [20]. Here, we found that our patient had SCLE with the presence of ANA and antiENA, such as anti-Ro/SSA, La/SSB, and Ro-52 antibodies. In addition, skin lesions only occurred after the initiation of teriflunomide intake. No other drugs or supplements were prescribed to the patient. Furthermore, a relapse event was observed after pausing hydroxychloroquine and tofacitinib citrate while the patient was on continuous teriflunomide treatment, ultimately strengthening the causal relationship between SCLE and teriflunomide.

Previous reports indicated that several DMT drugs have the potential of inducing autoimmune disease. For example, autoimmune thyroid events are common side effects after alemtuzumab [21] or interferon-beta [22] therapy in patients with MS. Furthermore, natalizumab [23] and interferon-beta [24] have been increasingly implicated as causes of drug-induced SCLE. These autoimmune diseases mostly occurred after using DMT for 2 to 5 months, which is consistent with our patient. The rare concurrence of SLE and MS has been described [25], and the possibility of a coincidental association cannot be ruled out. However, a study suggested that the interval between the two disease onsets should be at minimal one year or more [25], which did not apply to our patient. To date, the occurrence of SCLE and MS in the same individual has not been reported. Moreover, before using teriflunomide, our patient received a consultation with rheumatologists and underwent a structured examination, except for positive serum ANA, anti-Ro/SSA, La/SSB, and Ro-52 antibodies were positive, there were no other significant clinical symptoms and abnormal glandular function. Routine hematology and biochemistry were normal. Meanwhile, after using teriflunomide, skin lesions appeared on the face, and positive serum anti-Ro/SSA, anti-SSB, and anti-Jo-1, while other laboratory tests still were normal. The clinical and serological features were consistent with the diagnosis of SCLE. These results effectively excluded the possibility of concurrence of SCLE and MS.

Leflunomide and its active metabolite teriflunomide exert their therapeutic capability in MS by inhibiting pyrimidine synthesis. A pilot observational study indicated that leflunomide is beneficial for patients with SLE [26]. However, previously reported cases of new-onset SCLE associated with leflunomide therapy, indicated that patients in whom SCLE lesions precipitate or deteriorate after the initiation of leflunomide may represent a subset with a preexisting autoimmune diathesis [6]. Interestingly, serologic testing in our patient revealed that ANA positivity was also present prior to the treatment of teriflunomide. The temporal relationship between the onset of SCLE and the commencement of teriflunomide intake in this patient suggests the possibility that the latter may have been implicated in the development of the former. We further summarized the clinical features of our patient and those of previously reported cases of patients with leflunomide-induced SCLE [5–12]. The first leflunomide-induced SCLE case was reported in 2004 in a 59-year-old man with rheumatoid arthritis. His skin rash completely subsided without a further relapse after the discontinuation of leflunomide and treatment with oral hydroxychloroquine for 5 months [9]. Among our case and the previous leflunomide-induced SCLE cases (Table 1), 9/10 cases occurred in female adults (median age at 57, range 24–64; female to male ratio = 9:1). Skin manifestations quickly emerged after using leflunomide or teriflunomide for less than 6 months in 8/10 (80%) patients (median month at 2.5, range 0.75–11). Interestingly, we found that autoimmune antibodies existed in 9/10 (90%) patients, and 8/10 (80%) patients were positive for ANA, suggesting a direct causal relationship. Thus, we hypothesize that teriflunomide may act as an inducer of the emergence of SCLE, especially in female patients with preexisting autoimmune diathesis. This should be taken into consideration when prescribing teriflunomide as a treatment regime for MS. The side effect of teriflunomide-induced SCLE also should be considered when MS patients develop a rash after taking teriflunomide. Further investigations and controlled trials of the emergence of SCLE under treatment with teriflunomide are required.

**Table 1:** A summary of the clinical features of leflunomide-induced SCLE cases

**No./study**	**Patient**	**Gender/age**	**Primary disease**	**Dosage of leflunomide**	**Duration of using leflunomide (month)**	**SCLE manifestations**	**Autoimmune screening**	**Treatment (outcome)**
Gensburger D, et al. 2005 [7]	1	F/58	PSS	100 mg for 3 days, then 20 mg/d	1	Malar lupus skin reaction, leucopenia	ANA ( +), anti-ENA ( +), anti-SSA ( +), anti-SSB ( +)	Leflunomide stopped, steroids (eruption cleared)
Elias A, et al. 2005 [8]	2	F/64	RA	20 mg/d	4	Erythematous scaling plaques on the upper chest and arms	ANA ( +)	Leflunomide stopped, cholestyramine, prednisone (eruption resolved)
Chan S, et al. 2005 [10]	3	F/63	RA	NA	2	Multiple discrete annular and erythematous plaques on cheeks	ANA ( +), anti-Ro ( +)	Leflunomide stopped, corticosteroid (eruption resolved)
Goeb V, et al. 2006 [11]	4	F/59	RA	100 mg for 3 days, then 20 mg/d	12	Annular eruption on the back, neck and face	ANA ( +), anti-Ro ( +)	Leflunomide stopped, topical corticosteroids (eruption cleared)
Goeb V, et al. 2006 [11]	5	F/56	RA	100 mg for 3 days, then 20 mg/d	10	Papulosquamous eruption on the ears, upper back and cheeks	All antibodies negative	Leflunomide stopped (eruption improved)
Suess A, et al. 2008 [6]	6	F/42	RA	initiated at 100 mg/day, then 20 mg/d	3	Multiple annular erythemata, mouth and genital mucosa ulcers and crusts, leukopenia	ANA ( +), anti-Ro ( +)	Leflunomide stopped, colestyramine (eruption improved)
Singh H, et al. 2016 [5]	7	F/50	Hypothyroidism; seronegative RA	20 mg/d	1	Erythematous rash and annular lesions with crusted borders over face and anterior chest	ANA ( +), anti-Histone ( +), Anti-SSA ( +)	Leflunomide stopped, methotrexate, topical corticosteroids (eruption improved)
Kerr O, et al. 2004 [9]	8	M/59	RA	NA	1.5	Annular erythematous papules and plaques on the upper chest and arms	An-Ro ( +)	Leflunomide stopped, hydroxychloroquine (eruption subsided)
Marzano A, et al. 2008 [12]	9	F/24	AS	20 mg/d	0.75	Malar erythematous rash and erythematous annular lesions with crusted borders over the chest and abdomen	ANA ( +)	Leflunomide stopped, methylprednisolone (eruption resolved)

*Abbreviation*: *ANA* antinuclear antibodies, *AS* ankylosing spondylitis, *NA* not available, *PSS* primary Sjogren’s syndrome, *RA* rheumatoid arthritis, *SCLE* subacute cutaneous lupus erythematosus, *SLE* Systemic Lupus Erythematosus

We acknowledge several limitations in conclusively attributing the SCLE to teriflunomide. First, teriflunomide treatment did not discontinued to observe the progression of SCLE. Second, the current patient served as a single case, thus limiting statistical power on inferring a larger-scale population response. Additional research is needed to better evaluate the possible causal relationship between SCLE and teriflunomide. While rare, our report should serve as an exemplar for the cautious diagnosis of rare complications related to teriflunomide treatment and to consider SCLE as a potential adverse event when prescribing teriflunomide. Furthermore, the multi-disciplinary collaboration between neurology and dermatology is crucial for attaining the best therapeutic outcomes.

## Data Availability

All data generated or analyzed during this study are included in this published article.

## Abbreviations

DMT: Disease-modifying therapy
EDSS: Expanded disability status scale
MRI: Magnetic resonance imaging
MS: Multiple sclerosis
SCLE: Subacute cutaneous lupus erythematosus
SLE: Systemic lupus erythematosus
